# Preparing pets and their people: opportunity for veterinary teams to promote disaster preparedness in their communities

**DOI:** 10.3389/fvets.2025.1442482

**Published:** 2025-01-31

**Authors:** Joedy Quintana, Lindsey Viola, Valeria Sanchez, Danielle Scott, Colleen Duncan

**Affiliations:** College of Veterinary Medicine and Biomedical Sciences, Colorado State University, Fort Collins, CO, United States

**Keywords:** natural disaster, pets, preparedness, veterinary, public health, climate

## Abstract

Climate change has made disasters, and their associated health risks, more frequent and severe. Despite these growing risks, a substantial proportion of adults in the US do not have a disaster plan. Even for those who have disaster plans, it is unclear if these always include pets. The objective of this project was to explore the potential for veterinary teams to facilitate the development of pet-inclusive disaster plans through conversations during routine veterinary visits. We conducted two separate anonymous surveys, one for veterinary staff and one for veterinary clients. Overall, we found that both groups believe disasters are increasing and likely to impact people and their pets, however respondents remain largely unprepared for these events. Although both groups reported that the topic of disaster preparedness was not typically covered during veterinary visits, pet owners overwhelmingly agreed that pet health professionals are trustworthy sources of information, and that it would be helpful to have support from their veterinary team in developing a disaster plan that includes their pets. Barriers to such conversations, and potential solutions, were explored. Collectively these findings reinforce the role of veterinary professionals as trusted community members who can enhance public health and community resilience by integrating disaster preparedness into their practice.

## Introduction

Natural disasters occur when natural hazards intersect with vulnerable human populations and infrastructure, resulting in significant damage, disruption, and loss. In the United States, 2023 set a record for the highest number of billion-dollar disasters since 1980 (1) underscoring the significant and enduring impacts on the economy and human capital (2, 3). Strong scientific consensus shows that anthropogenic climate change is driving the rising frequency and intensity of extreme events such as heatwaves, heavy precipitation, droughts, and tropical cyclones (4). The World Meteorological Organization has reported a fivefold increase in weather-, climate- and water-related disasters from 1970 to 2019 (5). Beyond their physical and economic toll, these events often have substantial public health consequences (6, 7) with vulnerable cohorts such as children, the elderly, and the impoverished at particular risk of mental, emotional, and physical stress following disasters (8).

Companion animals are also impacted by these increasing disasters. If not safely evacuated by their caregivers, pets may be injured, killed, or lost. Disaster events can be sudden, such as the Marshall Fire in Colorado, in which more than a thousand pets perished within hours (9), or more protracted such Hurricanes Katrina and Rita, which impacted hundreds of thousands of dogs and cats (10). There are also long-term sequelae associated with animals in disasters, such as infectious disease outbreaks or feed and water contamination (1, 11). Disasters can also disrupt local veterinary infrastructure, complicating access to care and further complicating the animal health impacts (9). Finally, there are strong associations between keeping animals safe during disasters and improvements in public health outcomes more broadly (12) as people may fail to evacuate, or they re-enter disaster zones, to care for their animals (13, 14). In response to Katrina, Congress passed the Pets Evacuation and Transportation Standards Act of 2006 to provide authorization and resources for the inclusion of pets when planning for disasters (15–17). Opportunities to mitigate impacts on both animals and their caregivers would therefore have significant co-benefits (18).

Proactive planning and readiness, at both the individual and community level, are critical for mitigating the consequences of these events. Disaster preparedness significantly enhances public safety by minimizing casualties and injuries through timely and coordinated responses (19, 20). It also fosters community resilience and lessens the economic impact of disasters as prepared communities experience reduced damage to infrastructure and accelerated rehabilitation efforts (21, 22). Individual preparedness planning includes the development of evacuation plans for the home and workplace, rehearsing disaster plans, and procuring required/desired items in the event of a local disaster. Individuals who are more proactively prepared for a disaster tend to experience better outcomes across multiple scales (23). However, a 2022 FEMA study found less than half (45%) of US adults felt prepared for a disaster (24). For those with pets, preparedness planning should include contingencies for caring for their animals in the event of a disaster. Veterinarians are trusted community members who support public health efforts in addition to their animal responsibilities. The objective of this study was to explore the potential for veterinary teams (veterinarians, technicians, and clinic staff) to facilitate the development of a pet-inclusive disaster plan through conversations during routine veterinary visits.

## Materials and methods

Two anonymous surveys, described below, were developed, and administered through Qualtrics. Both surveys were classified as exempt by the Colorado State University Institutional Review Board.

### Veterinary clinic staff survey

This survey targeted employees of USA veterinary clinics over 18 years of age who consented to participate. There were 16 questions of mixed types including multiple choice, select all that apply, and ranking with some skip logic (Supplementary material 1). There were questions on respondent demographics, clinic type, beliefs about natural disasters, disaster preparedness planning for the clinic, and support and resources available for clients. For some questions, there was an opportunity for respondents to add additional responses if their answer was not included as a response option. All questions required a response except for the final open-ended opportunities to share disaster preparedness resource needs or additional feedback on the survey topic. The survey link was distributed through veterinary professional networks on social media, email, and by distribution of QR codes at professional networking events. The survey was open from July 3 to August 3, 2023. Survey participation was incentivized by offering those who completed the survey a chance to enter their name in a drawing for one of 10 $50 gift cards.

### Veterinary client survey

This survey targeted pet owners 18 years of age and older who live in the USA. Respondents were required to own a cat or dog, had utilized veterinary services within the past 2 years, and expressed consent to participate. The survey consisted of 12 multiple choice and select all that apply questions with some skip logic (Supplementary material 2). There were questions on respondent demographics, beliefs about natural disasters and disaster preparedness planning, with an emphasis on their relationship with veterinary teams. One attention check question was also included. All questions required a response except for the final open-ended question, which provided an opportunity for people to provide any additional information they felt relevant. Veterinary clients were identified through Amazon Mechanical Turk. Survey participants received $1 for completing the questionnaire. The survey was available from July 18 to September 11, 2023.

### Data analysis

Descriptive and comparative statistics were conducted using SPSS. When present, free text responses were categorized into broad themes based on common sentiments shared by survey respondents. Briefly, fill in responses to “other _____” or final open text comments were reviewed by multiple authors (JQ, CD) who independently looked for shared elements from which a series of thematic categories were identified. The frequency of these open text responses was reported in rank order.

## Results

### Veterinary clinic staff survey

A total of 774 respondents participated in the survey. Respondents were from 47 states (Supplementary Table 1) and represented a variety of roles at the veterinary clinic including veterinarians (43%), veterinary technicians (26%), veterinary assistants (15%), practice managers (8%), receptionists (4%) or other (4%), including veterinary students, inventory managers, and kennel assistants. The majority of respondents worked in small animal practices (86%) with a small number in mixed (9%), large (3%), or “other” (2%) which included exotics, wildlife, shelters, and academia. Most respondents were in suburban communities (62%) followed by urban (21%) then rural (17%) communities.

**Figure 1:**
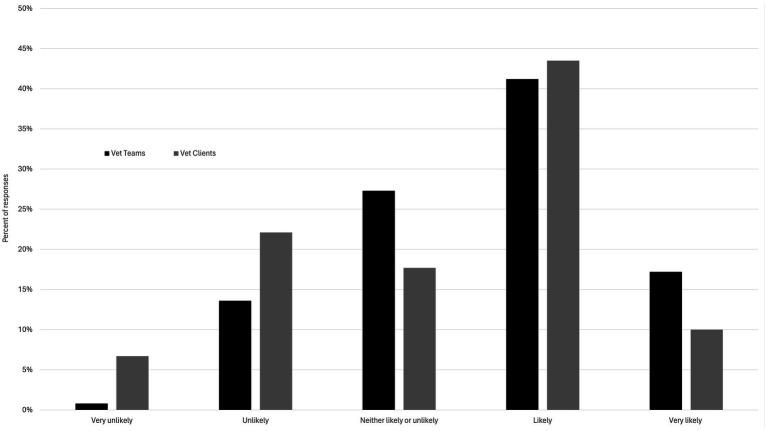
Client and veterinary clinic staff perceptions regarding the likelihood of them, and their pets, being affected by a disaster in the next 10 years.

Respondents were asked about the frequency of disasters occurring now relative to 10 years ago, and most respondents felt they were slightly more (46%) or much more (33%) frequent now. Only 19% reported disasters to be neither more or less frequent now, and those indicating disasters to be slightly less or much less frequent were 1% of the respondent pool each. The majority of respondents (59%) indicated they are likely or very likely to be affected by a disaster in the next ten years (Figure 1).

Most (57%) respondents indicated they had not received any training about preparedness for animals in disasters. When asked if there is a plan in place for their veterinary clinic in the event of a disaster, the majority responding “no” (36%) or “I do not know” (29%). Of the 35% responding that their clinic has a disaster plan, the majority reported never practicing it (39%) followed by annual practice (37%), practice more than every 2 years (11%), practice every 2 years (8%), or practicing every 6 months (5%). Additionally, these respondents indicated disaster plans were most commonly updated annually (38%) followed by never (21%), more than 2 years (22%), every 2 years (15%), or every 6 months (4%). Clinic disaster plans were most often reviewed with staff/employees annually (50%) followed by never (19%), >2 years (16%), every 6 months (8%) or every 2 years (7%). Where disaster plans were in place, most (60%) were reviewed by new employees during onboarding.

When asked how often disaster preparedness is discussed with clients during routine visits, most (57%) respondents answered “never” followed by “rarely” (32%), and “sometimes” (9%). Only 2% reported “very often” or “always” discussing disaster preparedness with clients during routing visits. The most frequently selected barriers to discussing disaster preparedness with clients were, in ranked order: (1) veterinary teams are not sure of how they should be preparing their clients for disasters (35%); (2) natural disasters are not common in the area where the veterinary team practices (29%); (3) the veterinary team is not sure what their role is in disaster preparedness (21%); (4) respondents do not face barriers (8%); and (5) disaster preparedness is a difficult subject to discuss (7%). Additional barriers, provided by respondents through write-in responses, indicated clients’ lack of interest or belief that natural disasters will not affect them (*n* = 8); respondents had never thought to discuss the topic of natural disasters preparedness (*n* = 9); respondents work in a specialty area like oncology or emergency where they do not interact with clients (*n* = 19); and limited time during client visits (*n* = 41).

Only a small number (13%) of respondents answered that they have resources, such as handouts, checklists, and websites, available for their clients to guide their development of pet-inclusive disaster plans. Respondents affirmed that the following would be helpful or very helpful: (a) resources (e.g., brochures, pamphlets, online material) for pet owners to create a disaster plan (87%); (b) resources (e.g., protocols, guidelines) for optimizing a clinic’s disaster plan (85%); (c) courses about preparing for disasters (77%); and (d) courses about how to discuss disaster preparedness with your client (72%). Other helpful resources indicated by respondents through write-in answers included online continuing education, checklists that are easy and accessible for clients, social media resources, geographic specific disaster preparedness information, and a website to provide clients with that includes valuable information. Finally, there was an option for respondents to share anything else they desired about the survey or disaster preparedness. There were two commonly shared sentiments; the first is the benefit of regionally specific information, and the second was an interest in how veterinary teams could increase awareness and concern of clients regarding natural disasters. Several respondents reported it is difficult to get clients to care about natural disaster preparedness and that many clients believe that a natural disaster will never happen to them. Many of the respondents shared that they appreciated the research project and were glad that natural disaster preparedness was being discussed in the veterinary field.

### Veterinary client survey

There were 299 veterinary clients who consented to participate and correctly answered the attention check question included in the final analysis. Respondents were from 41 states (Supplementary Table 1), and predominantly from suburban areas (54%) with the remaining equally split from urban (23%) and rural areas (23%).

Regarding the frequency of disasters occurring now relative to 10 years ago, most respondents felt they occur slightly more (46%) or much more frequent now (33%), as compared to neither more or less frequent now (18%) or slightly less frequent now (3%). No respondents selected that disasters are much less frequent now than 10 years ago. Regarding the “likelihood that you and your pet(s) will be affected by a disaster in the next 10 years,” most respondents answered in the affirmative “likely” or “very likely” (figure 1).

Respondents were asked about their current disaster plan, and most reported they do not have a current plan but have considered forming one (46%), followed by those who have not considered forming a disaster plan (21%), and those who have a current disaster plan which needs to be updated (20%). Only 13% of respondents indicated they have a current disaster plan, with all affirming their plan includes their pets.

Respondents indicated that pet health professionals were “trustworthy” (55%) or “very trustworthy” (28%) in guiding the development of disaster plans that includes pets, with far less indicating neutral (15%), and only a small number (2%) selecting “untrustworthy” or “extremely untrustworthy.” Regarding the frequency of someone at their veterinary clinic discussing disaster plans that include their pets, only 5% selected “yes.” When asked how helpful they would find their veterinary clinic’s support to be in developing a disaster plan that includes their pet(s), more than three-quarters of responses were positive (“helpful” = 58% or “very helpful” = 18%) followed by neutral (21%) and a small number of negative responses (unhelpful = 3%). Of the list of options provided regarding information clients could receive from veterinary clinics, the highest-ranking response was (a) how to prepare pets for disasters (37%), followed by (b) worksheets for developing a plan for pets (27%), (c) how disasters affect pets (19%), and (d) the type of disasters that occur in a specific region (16%). Other information clients would like from their veterinary clinic (provided by write-in responses) included what to do during a disaster in terms of boarding facilities or places to house pets, how to handle prescriptions, and what to do if owners are separated from their pet. Finally, there was an option for respondents to share anything else they would like to add about this survey or disaster preparedness. Some of the major themes that respondents wanted included resources about regionally specific information, checklists of what clients should have prepared in case of an emergency, a list of safe places to take pets during a natural disaster, and how to handle stressed animals during a disaster.

## Discussion

Results of these surveys highlighted challenges and opportunities shared between veterinary clinic staff and their clients, making it pragmatic to discuss their perspectives together. Despite strong agreement by both veterinary staff and clients that disasters are becoming more frequent, and with over half of all respondents expressing the likelihood of them and their pet being affected by a disaster in the next 10 years, our results suggest that both groups are largely unprepared for such events. This is consistent with patterns and projections of disasters and highlights several opportunities for veterinary staff to bolster disaster preparedness in communities through their own actions and as trusted community leaders and educators.

Being able to maintain climate resilient veterinary health systems, those capable of withstanding the disasters already known to disrupt physical and social infrastructure, is one of three critical areas in which the health sector must act on climate (25). In our survey, only 35% of veterinary respondents reported their clinic had an up-to-date clinic disaster plan. This is less than a similar, but smaller (*n* = 209), study conducted on veterinarians practicing in Mississippi, where 43% have a disaster plan (26) which may be explained by people in the southeast being generally more prepared (27) or the fact that the majority of those surveyed in Mississippi had already experienced a disaster making them more likely to have a disaster plan. In addition to creating a clinic disaster plan, it is essential to conduct frequent exercises and practice drills such as safe building evacuation or sheltering in place (28). In our survey, of the 35% of clinics with disaster plans, 37% reported never practicing the plan, 21% reported never updating the plan, and 18% reported never reviewing the plan with their staff, effectively nullifying the disaster preparedness effort.

To mitigate disruption of veterinary health services in the face of disaster, it is essential that that the profession expand emergency preparedness efforts. At the clinic level, this centers around the development of a clinic specific disaster plan and ensuring that the entire team is up to date with the plan. Several resources exist to aid in the planning, both within veterinary networks and beyond the profession. For example, in the US, the American Veterinary Medical Association provides resources for members on “Emergency Planning for Veterinary Practices” (29), a workbook on “How to Write a Disaster Plan for your Clinic” (30) and more in-depth continuing education courses on “Disaster and Business Continuity Planning” (30). Similarly, the World Organization for Animal Health has published “Guidelines on Disaster Management and Risk Reduction in Relation to Animal Health and Welfare and Veterinary Public Health” (31). A disaster plan should be viewed as a “living document” and reviewed and updated regularly. It should also be practiced routinely with staff, both during planned practices where time is blocked off in the schedule, as well as initiating unannounced practices during a lull or on a slow day. All employees should be introduced to the plan during onboarding and given ample opportunity to ask questions or suggest revisions to the plan should they see opportunities for improvement. As clinic teams work to develop, practice, and refine preparedness plans at the clinic, they should also support employees to develop their own household plan that includes their pets.

Encouraging household, and pet, preparedness planning need not be restricted to staff. In our survey, veterinary clients are similarly unprepared despite their acknowledgement that disasters are increasing in frequency and have the potential to impact their pets. While one third of clients responded that they have a plan, only 13% reported that their plan was up to date. This is similar to, albeit lower than, a 2021 survey reported by ASPCA that says 46% of pet owners have a plan in place (32). This difference may reflect variability in respondent geography, lived experience, or differences in question framing as we explicitly asked if the plan was up to date. With ~87 million pet owning households in the USA alone (33), even if half had a plan, that leaves more than 40 million without, and therefore an opportunity for veterinary teams to expand their efforts to build resilience in their communities.

Veterinarians have a long history in safeguarding public health through their expertise in disease control, zoonotic disease surveillance, food safety regulation, and the promotion of animal welfare. Expanding into disease preparedness, particularly for animal caregivers, is well within the scope of professional practice. The public generally has a positive perception of veterinarians, even more favorable than physicians, specifically finding them more approachable, sensitive, and understanding (34). Results of this survey also support veterinarians as trustworthy, with the overwhelming majority responding that they trust pet health professionals to help them develop a disaster plan that includes their pets. By raising awareness of disaster preparedness and sharing resources with their clients, veterinary professionals will expand their public health purview by building disaster resilience, that is the ability to recover quickly, in their communities.

Despite client interest in learning more about disaster preparedness at the veterinary clinic, both clients and veterinary teams reported this topic was very rarely discussed at the veterinary clinic. In this study, we identified several barriers to engagement on the topic, some of which are resolvable with relatively minimal disruption. To address the reported lack of knowledge about how to prepare people and their pets for disasters, we could better connect clinic staff with existing resources on the topic, such as those from national associations like the AVMA (35) or FEMA (36), which have client checklists and other helpful tools. Our survey work also highlighted a need for access to regionally specific resources, such information on disasters more common in an area or local options for sheltering animals. Such resources may already be available through state or regional veterinary medical associations, or local clinics could work together to develop information that could be disseminated through both veterinary networks as well as regional disaster management groups.

Respondents also reported lack of time during veterinary visits as a barrier to disaster information sharing. Some ways to share this information without increasing workload include passive strategies such as sharing prepared materials with new clients as part of a broader resource packet or having posters with QR codes linked to existing resources in waiting rooms. Alternatively, more active strategies could include asking if the client has updated their disaster preparedness plan as part of routine wellness visits and providing resources if they have not. Providing a variety of opportunities for clients to engage with available resources will help to normalize discussion of the topic which can benefit both pets and people. It is also important to note that this engagement need not be restricted to veterinary-client interactions. Sharing of preparedness resources could take place at any client contact points during the visit by engaging veterinary technicians and other members of the staff. Finally, some people find disaster preparedness a difficult topic to discuss. We presumed that this may be due to political barriers, most notably in the USA (37), given the association between disasters and climate change, however, research consistently supports that framing climate change as a health issue reduces this polarization, raises awareness and urgency by making its impacts more relatable, and better mobilizes public support for climate action (38–40).

There are several limitations to this survey-based study, highlighting the need for additional research in on the topic. The veterinary clinic staff survey was opportunistically distributed and, based on the respondent demographics, geographically biased. Similarly, the veterinary client survey lacked representation from some geographic regions and, while Amazon’s Mechanical Turk platform has been shown to be more representative of the US population than convenience sampling, there is typically an over-representation of younger and urban based respondents (41, 42). Respondents’ previous experience with disasters, which was not accounted for in this study, may have influenced their decision to participate in the project and their responses to individual questions. Finally, the lack of shared client-veterinary relationships makes it impossible to draw conclusions about specific interactions between the two survey groups.

The focus of this study was on expanding disaster preparedness at veterinary clinics; however, it should be noted that there are many more ways for animal health professionals to support disaster preparedness, response, and recovery efforts in their communities. Previous research has similarly identified opportunities to better equip veterinary teams to respond in disaster situations (43), suggesting there may be a broader need for expanding training to animal health professionals on this topic. Future research on the most effective ways to engage veterinary clients in veterinary clinics could be helpful to inform best practices in communication and dissemination of the information. Given the projected increase in disasters in the USA and beyond (4) integrating disaster preparedness planning into our scope of preventive care could go a long way to protecting the health of both pets and their people.

## Data Availability

The original contributions presented in the study are included in the article/Supplementary material, further inquiries can be directed to the corresponding author.

## References

[1] (NCEI) NNCfEI. (2024). Billion-Dollar weather and climate disasters. Available at: https://www.ncei.noaa.gov/access/billions/ (Accessed October 15, 2024)

[2] Opper IM Park RJ Husted L. The effect of natural disasters on human capital in the United States. Nat Hum Behav. (2023) 7:1442–53. doi: 10.1038/s41562-023-01610-z, PMID: 37264085

[3] Boustan LP Kahn ME Rhode PW Yanguas ML. The effect of natural disasters on economic activity in US counties: a century of data. J Urban Econ. (2020) 118:103257. doi: 10.1016/j.jue.2020.103257

[4] Seneviratne SIX Zhang MA Badi W Dereczynski C Di Luca A Ghosh S. Weather and climate extreme events in a changing climate In: Masson-Delmotte V Zhai P Pirani A Connors SL Péan C Berger S, editors. Climate change 2021: The physical science basis contribution of working group I to the sixth assessment report of the intergovernmental panel on climate change. Cambridge: Cambridge University Press (2021). 1513–766.

[5] WMO. WMO atlas of mortality and economic losses from weather, climate and water extremes (1970–2019). Geneva: WMO (2021).

[6] Noji EK. The public health consequences of disasters. Prehosp Disaster Med. (2000) 15:21–31. doi: 10.1017/S1049023X00025255 11227602

[7] Noji EK. Public health issues in disasters. Crit Care Med. (2005) 33:S29–33. doi: 10.1097/01.CCM.0000151064.98207.9C, PMID: 15640676

[8] Benevolenza MA DeRigne L. The impact of climate change and natural disasters on vulnerable populations: a systematic review of literature. J Hum Behav Soc Environ. (2019) 29:266–81. doi: 10.1080/10911359.2018.1527739

[9] Irvine L Andre C. Pet loss in an urban firestorm: grief and Hope after Colorado’s Marshall fire. Animals. (2023) 13:416. doi: 10.3390/ani13030416, PMID: 36766306 PMC9913112

[10] Wittnich C Belanger M. How is animal welfare addressed in Canada's emergency response plans? J Appl Anim Welf Sci. (2008) 11:125–32. doi: 10.1080/10888700801925976, PMID: 18444033

[11] Levy JK Lappin MR Glaser AL Birkenheuer AJ Anderson TC Edinboro CH. Prevalence of infectious diseases in cats and dogs rescued following hurricane Katrina. J Am Vet Med Assoc. (2011) 238:311–7. doi: 10.2460/javma.238.3.311, PMID: 21281213

[12] Thompson K. Facing disasters together: how keeping animals safe benefits humans before, during and after natural disasters. Rev Sci Tech. (2018) 37:223–30. doi: 10.20506/rst.37.1.2753, PMID: 30209416

[13] Hall MJ Ng A Ursano RJ Holloway H Fullerton C Casper J. Psychological impact of the animal-human bond in disaster preparedness and response. J Psychiatr Pract. (2004) 10:368–74. doi: 10.1097/00131746-200411000-00005, PMID: 15583518

[14] Heath SE Kass PH Beck AM Glickman LT. Human and pet-related risk factors for household evacuation failure during a natural disaster. Am J Epidemiol. (2001) 153:659–65. doi: 10.1093/aje/153.7.659, PMID: 11282793

[15] Hunt M Al-Awadi H Johnson M. Psychological sequelae of pet loss following hurricane Katrina. Anthrozoös. (2008) 21:109–21. doi: 10.2752/175303708X305765

[16] Zottarelli LK. Broken bond: an exploration of human factors associated with companion animal loss during hurricane Katrina1. Sociol Forum. (2010) 25:110–22. doi: 10.1111/j.1573-7861.2009.01159.x

[17] Evacuation P. Transportation Standards Act (2006). Available at: https://www.congress.gov/bill/109th-congress/house-bill/3858

[18] Chadwin R. Evacuation of pets during disasters: a public health intervention to increase resilience. Am J Public Health. (2017) 107:1413–7. doi: 10.2105/AJPH.2017.303877, PMID: 28727532 PMC5551593

[19] Sandifer PA Walker AH. Enhancing disaster resilience by reducing stress-associated health impacts. Frontiers. Public Health. (2018) 6:373. doi: 10.3389/fpubh.2018.00373, PMID: 30627528 PMC6309156

[20] Khatri RB Endalamaw A Erku D Wolka E Nigatu F Zewdie A. Preparedness, impacts, and responses of public health emergencies towards health security: qualitative synthesis of evidence. Arch Public Health. (2023) 81:208. doi: 10.1186/s13690-023-01223-y, PMID: 38037151 PMC10687930

[21] Kapucu N. Collaborative emergency management: better community organising, better public preparedness and response. Disasters. (2008) 32:239–62. doi: 10.1111/j.1467-7717.2008.01037.x, PMID: 18380853

[22] Johnston KA Taylor M Ryan B. Engaging communities to prepare for natural hazards: a conceptual model. Nat Hazards. (2022) 112:2831–51. doi: 10.1007/s11069-022-05290-2

[23] Dodd W Scott P Courtney H Scott C Rose C Cunsolo A. Lived experience of a record wildfire season in the Northwest Territories, Canada. Can J Pub Health. (2018) 109:327–37. doi: 10.17269/s41997-018-0070-5, PMID: 29981098 PMC6964492

[24] FEMA. National Household Survey on disaster preparedness (NHS), key findings | individual and community preparedness division 2023. Washington, DC: FEMA (2022).

[25] Viola L Quintana J Sanchez V Scott D Griffenhagen GM Duncan C. Veterinary anesthesia: an opportunity to reduce the environmental footprint of clinical care. J Am Vet Med Assoc. (2024) 22:1–8. doi: 10.2460/javma.24.01.0059 38688307

[26] Huston CL Ebers KL. Assessing disaster preparedness and educational needs of private veterinary practitioners in Mississippi. J Vet Med Educ. (2020) 47:230–8. doi: 10.3138/jvme.0618-074r, PMID: 31194634

[27] Cook F Howe P. Geographic variation in household disaster preparedness in the United States. Ann Am Assoc Geogr. (2024) 114:314–33. doi: 10.1080/24694452.2023.2271560

[28] Chartoff SE Kropp AM Roman P. Disaster Planning. In: StatPearls [Internet]. Treasure Island (FL): StatPearls Publishing (2025). 29261948

[29] Association AVM. (2024). Emergency planning for veterinary practices. Available at: https://www.avma.org/resources-tools/animal-health-and-welfare/disaster-preparedness/emergency-planning-veterinary-practices (Accessed October 15, 2024)

[30] Association AVM. (2024). Disaster preparedness. Available at: https://www.avma.org/resources-tools/animal-health-and-welfare/disaster-preparedness (Accessed October 15, 2024)

[31] WOAH. Guidelines on disaster management and risk reduction in relation to animal health and welfare and veterinary public health. Paris: WOAH (2016).

[32] ASPCA. ASPCA survey shows 83% of pet owners are impacted by disasters, fewer than half have preparedness plans. New York, NY: ASPCA (2021).

[33] Association APP. (2024). Industry trends and stats. Available at: https://www.americanpetproducts.org/research-insights/industry-trends-and-stats (Accessed October 15, 2024)

[34] Kedrowicz AA Royal KD. A comparison of public perceptions of physicians and veterinarians in the United States. Vet Sci. (2020) 7:50. doi: 10.3390/vetsci7020050, PMID: 32331215 PMC7357132

[35] Association AVM. (2024). Pets and Disasters. Available at: https://www.avma.org/resources-tools/pet-owners/emergency-care/pets-and-disasters.

[36] FEMA. (2023). Prepare your pets for disasters. Available at: https://www.ready.gov/pets (Accessed October 15, 2024)

[37] Kramer CG McCaw KA Zarestky J Duncan CG. Veterinarians in a changing global climate: educational disconnect and a path forward. Frontiers in veterinary. Science. (2020) 7:613620. doi: 10.3389/fvets.2020.613620, PMID: 33392298 PMC7773640

[38] Myers TA Nisbet MC Maibach EW Leiserowitz AA. A public health frame arouses hopeful emotions about climate change. Clim Chang. (2012) 113:1105–12. doi: 10.1007/s10584-012-0513-6, PMID: 39831055

[39] Alame D Truog RD. How should clinicians weigh the benefits and harms of discussing politicized topics that influence their individual Patients' health? AMA J Ethics. (2017) 19:1174–82. doi: 10.1001/journalofethics.2017.19.12.ecas3-1712, PMID: 29278343

[40] Adlong W Dietsch E. Environmental education and the health professions: framing climate change as a health issue. Environ Educ Res. (2015) 21:687–709. doi: 10.1080/13504622.2014.930727

[41] Berinsky AJ Huber GA Lenz GS. Evaluating online labor markets for experimental research: Amazon. com's mechanical Turk. Polit Anal. (2012) 20:351–68. doi: 10.1093/pan/mpr057

[42] Huff C Tingley D. “Who are these people?” evaluating the demographic characteristics and political preferences of MTurk survey respondents. Res Politics. (2015) 2:2053168015604648. doi: 10.1177/2053168015604648

[43] Ablah E Benson LN Tinius AM Gebbie KM. Assessment of emergency preparedness of veterinarians in New York. J Vet Med Educ. (2009) 36:122–7. doi: 10.3138/jvme.36.1.122, PMID: 19435999

