# Supplementary material for: Preparing pets and their people: opportunity for veterinary teams to promote disaster preparedness in their communities
Source: Front Vet Sci. 2025 Jan 31;12:1442482. doi: 10.3389/fvets.2025.1442482 (PMC11825779; doi:10.3389/fvets.2025.1442482)
Supplement: Supplementary file 1 [file Supplementary_file_1.docx]

Supplemental Material 1: Survey Questions for Veterinary Clinic Staff

What state do you currently work in?

▼ Alabama ... Wyoming

Which of the following best describes your role at the veterinary clinic?

- Receptionist
- Veterinary Technician (Registered/Certified/Licensed)
- Veterinary Assistant
- Veterinarian
- Practice Manager
- Other __________________________________________________

Which of the following best describes the type of clinic you work in?

- Small animal
- Large animal
- Mixed animal
- Other __________________________________________________

How would you describe the community where you live?

- Urban
- Suburban
- Rural

**For the following questions we are referring to 'natural' disasters, which are events such as:  heatwaves, flooding, hurricanes, earthquakes, and wildfires.**

Which of the following best describes your belief regarding the frequency of disasters now, relative to ten years ago?

- Much more frequent now
- Slightly more frequent now
- Neither more or less frequent now
- Slightly less frequent now
- Much less frequent now

Please rate the likelihood that you and your pet(s) will be affected by a disaster in the next ten years.

- Very likely
- Likely
- Neither likely or unlikely
- Unlikely
- Very unlikely

Have you received any training about preparedness for animals in disasters?

- Yes
- No

Is there a plan in place for your veterinary clinic in the event of a disaster?

- Yes
- No
- I don't know

Skip To: Q12 If Is there a plan in place for your veterinary clinic in the event of a disaster? = No

Skip To: Q12 If Is there a plan in place for your veterinary clinic in the event of a disaster? = I don't know

Please answer the following questions based off of your disaster plan.

|  | Every 6 months | Every year | Every 2 years | >2 years | Never |
| --- | --- | --- | --- | --- | --- |
| How often do you practice your clinic disaster plan? |  |  |  |  |  |
| How often do you update your clinic disaster plan? |  |  |  |  |  |
| How often do you review your clinic disaster plan with your staff/employees? |  |  |  |  |  |

Do new employees review the disaster plan during onboarding?

- Yes
- No

How often do you or someone at your clinic discuss disaster preparedness with your clients during routine visits?

- Always
- Very often
- Sometimes
- Rarely
- Never

Skip To: Q14 If How often do you or someone at your clinic discuss disaster preparedness with your clients during... = Always

What are some barriers you face in discussing disaster preparedness with your clients? *(Select all that apply)*

- Natural disasters are not common in the area where I practice
- It is a difficult subject to discuss with clients
- I am not sure what my role is in disaster preparedness
- I am not sure how I should be preparing clients for disasters
- I do not face barriers in discussing disaster preparedness with my clients
- Other __________________________________________________

Do you have resources (eg. handouts, checklist, websites) available for your clients to help them develop disaster plans that include pets?

- Yes
- No
- I don't know

Please indicate how helpful the following resources on disaster preparedness would be for your clinic.

|  | Very helpful | Somewhat helpful | Neutral | Not helpful | Very unhelpful |
| --- | --- | --- | --- | --- | --- |
| Courses about preparing for disasters |  |  |  |  |  |
| Courses about how to discuss disaster preparedness with your client |  |  |  |  |  |
| Resources (e.g. protocols, guidelines) for optimizing a clinic's disaster plan |  |  |  |  |  |
| Resources (e.g. brochures, pamphlets, online material) for pet owners to create a disaster plan |  |  |  |  |  |

Are there any other resources on disaster preparedness that would be helpful for your clinic that were not mentioned above? *(optional)*

________________________________________________________________

Is there anything else you would like to add about this survey or disaster preparedness? *(optional)*

________________________________________________________________

| Page Break |  |
| --- | --- |
