# Supplementary material for: Preparing pets and their people: opportunity for veterinary teams to promote disaster preparedness in their communities
Source: Front Vet Sci. 2025 Jan 31;12:1442482. doi: 10.3389/fvets.2025.1442482 (PMC11825779; doi:10.3389/fvets.2025.1442482)
Supplement: Supplementary file 2 [file Supplementary_file_2.docx]

Supplemental Material 2: Survey Questions for Veterinary Clients

What state do you currently reside in?

▼ Alabama ... Wyoming

How would you describe the community where you live?

- Urban
- Suburban
- Rural

**For the following questions we are referring to 'natural' disasters, which are events such as: heatwaves, flooding, hurricanes, earthquakes, and wildfires.**

Which of the following best describes your belief regarding the frequency of disasters now, relative to ten years ago?

- Much more frequent now
- Slightly more frequent now
- Neither more or less frequent now
- Slightly less frequent now
- Much less frequent now

Please rate the likelihood that you and your pet(s) will be affected by a disaster in the next ten years.

- Very likely
- Likely
- Neither likely or unlikely
- Unlikely
- Very unlikely

Please choose the statement below that best describes your current disaster plan.

- I have a current disaster plan
- I have a current disaster plan, but it needs to be updated
- I do not have a current disaster plan but have considered forming a disaster plan
- I have not considered forming a disaster plan

Skip To: Q8 If Please choose the statement below that best describes your current disaster plan. = I have a current disaster plan

Skip To: Q8 If Please choose the statement below that best describes your current disaster plan. = I have a current disaster plan, but it needs to be updated

Skip To: Q9 If Please choose the statement below that best describes your current disaster plan. = I do not have a current disaster plan but have considered forming a disaster plan

Skip To: Q9 If Please choose the statement below that best describes your current disaster plan. = I have not considered forming a disaster plan

Does your disaster plan include your pets?

- Yes
- No

Managing pets in disasters needs people paying attention. Please choose 'somewhat agree' if you are paying attention.

- Strongly agree
- Somewhat agree
- Neither agree or disagree
- Somewhat disagree
- Strongly disagree

Please indicate your level of trust in pet health professionals (Veterinarian, Veterinary Technicians, Veterinary Nurses, Veterinary Behaviorists) to guide you in the development of disaster plans that include your pets.

- Very trustworthy
- Trustworthy
- Neutral
- Untrustworthy
- Extremely untrustworthy

Has someone at your veterinary clinic discussed disaster plans that include your pet?

- Yes
- No

How helpful would you find your veterinary clinic’s support to be in developing a disaster plan that includes your pet(s)?

- Very helpful
- Helpful
- Neutral
- Unhelpful
- Very unhelpful

What information would you like to receive from your veterinary clinic to
 inform you on how to prepare your pet(s) for a natural disaster? *Select all that apply.*

- How disasters affect pets
- The type of disasters that occur in your area
- How to prepare pets for disasters
- Worksheet for developing a plan for your pets
- Other __________________________________________________

Is there anything else you would like to add about this survey or disaster preparedness and your pet? *(Optional)*

________________________________________________________________

| Page Break |  |
| --- | --- |
