# Supplementary material for: Preparing pets and their people: opportunity for veterinary teams to promote disaster preparedness in their communities
Source: Front Vet Sci. 2025 Jan 31;12:1442482. doi: 10.3389/fvets.2025.1442482 (PMC11825779; doi:10.3389/fvets.2025.1442482)
Supplement: Supplementary file 3 [file Supplementary_file_3.docx]

**Supplemental Material 3, Table 1: Number of respondents, by state, to the veterinary clinic staff and veterinary client surveys**

| **State** | **Veterinary Clinic Staff** | **Veterinary Clients** |
| --- | --- | --- |
| Alabama | 14 | 5 |
| Alaska | 4 |  |
| Arizona | 23 | 5 |
| Arkansas | 5 |  |
| California | 59 | 26 |
| Colorado | 84 | 7 |
| Connecticut | 5 | 2 |
| Delaware | 1 | 1 |
| Florida | 45 | 25 |
| Georgia | 10 | 15 |
| Hawaii | 3 | 2 |
| Idaho | 4 |  |
| Illinois | 23 | 10 |
| Indiana | 17 | 3 |
| Iowa | 9 |  |
| Kansas | 4 |  |
| Kentucky | 3 | 8 |
| Louisiana | 11 | 2 |
| Maine | 7 | 1 |
| Maryland | 12 | 3 |
| Massachusetts | 21 | 4 |
| Michigan | 39 | 21 |
| Minnesota | 10 | 6 |
| Mississippi |  | 6 |
| Missouri | 12 | 6 |
| Montana | 6 | 1 |
| Nebraska | 6 | 2 |
| Nevada | 8 | 2 |
| New Hampshire | 3 | 3 |
| New Jersey | 16 | 6 |
| New Mexico | 10 | 1 |
| New York | 37 | 12 |
| North Carolina | 26 | 11 |
| North Dakota | 2 |  |
| Ohio | 16 | 24 |
| Oklahoma | 10 | 2 |
| Oregon | 15 | 3 |
| Pennsylvania | 31 | 28 |
| South Carolina | 15 | 2 |
| South Dakota | 2 |  |
| Tennessee | 19 | 11 |
| Texas | 52 | 10 |
| Utah | 5 | 2 |
| Vermont | 5 |  |
| Virginia | 28 | 4 |
| Washington | 24 | 3 |
| West Virginia |  | 2 |
| Wisconsin | 10 | 11 |
| Wyoming | 3 | 1 |
| **Total** | **774** | **299** |
